# PEG/PEI-functionalized single-walled carbon nanotubes as delivery carriers for doxorubicin: synthesis, characterization, and in vitro evaluation

**DOI:** 10.3762/bjnano.11.155

**Published:** 2020-11-13

**Authors:** Shuoye Yang, Zhenwei Wang, Yahong Ping, Yuying Miao, Yongmei Xiao, Lingbo Qu, Lu Zhang, Yuansen Hu, Jinshui Wang

**Affiliations:** 1 College of Bioengineering, Henan University of Technology, Zhengzhou, P. R. China; 2 College of Chemistry and Chemical Engineering, Henan University of Technology, Zhengzhou, P. R. China; 3 College of Chemistry and Molecular Engineering, Zhengzhou University, Zhengzhou, P. R. China

**Keywords:** antitumor activity, cellular uptake, PEG functionalization, PEI functionalization, poly(ethylene glycol) (PEG), polyethylenimine (PEI), single-walled carbon nanotubes

## Abstract

Single-walled carbon nanotubes (SWCNTs) have attracted great interest regarding drug-delivery applications. However, their application has been limited by some inherent disadvantages. In this study, raw SWCNTs were purified with different oxidizing acids, and the resulting shortened CNTs were conjugated with poly(ethylene glycol) (PEG) and polyethylenimine (PEI). The different nanocarriers, that is, CNTs-COOH (CNTs), CNTs-PEG and CNTs-PEG-PEI, were systematically characterized and evaluated in terms of drug loading, in vitro release, cytotoxicity towards MCF-7 cells and cellular uptake. The results showed that all CNT carriers had a high drug loading capacity. In comparison with CNTs-COOH and CNTs-PEG, CNTs-PEG-PEI showed a more rapid drug release under acidic conditions and a higher antitumor activity. Furthermore, fluorescence detection and flow cytometry (FCM) analysis results indicated that the internalization into cells of CNTs-PEG-PEI was significantly enhanced, thus inducing tumor cell death through apoptosis more efficiently. The above series of benefits of CNTs-PEG-PEI may be attributed to their good dispersibility and comparably higher affinity to tumor cells due to the difunctionalization. In summary, the PEG- and PEI-conjugated CNTs may be used as novel nanocarriers and the findings will contribute to the rational design of multifunctional delivery vehicles for anticancer drugs.

## Introduction

To date, chemotherapy is the most common therapy for cancer treatment. However, the inability of chemotherapeutic agents to distinguish cancer cells from normal cells due to nonspecific distribution and lack of selectivity results in severe toxic side effects for health [1–2]. Therefore, a variety of nanoscale delivery systems have been designed for the controlled release of chemotherapy drugs to decrease the distribution in normal tissues, and to improve the biological half-lives [3–6].

Carbon nanotubes (CNTs) have attracted great interest for biomedical applications, including the delivery of bioactive molecules such as drugs, the targeted cancer therapy, and biological imaging, because of their unique properties, including large surface area, relatively low density, high stability and other inherent mechanical, optical and electrical properties [7–9]. CNTs can be produced as single-walled CNTs (SWCNTs) or multi-walled CNTs (MWCNTs) [10]. SWCNTs can be easily synthesized and efficaciously traverse biological barriers [11–13]. Despite a series of advantages, the practical application of CNTs has been restricted by a number of drawbacks, such as high hydrophobicity and rapid aggregation in aqueous media, which are associated with cytotoxicity and other harmful cellular effects [14–18]. Numerous attempts and efforts have been devoted to improve the dispersibility in water and to reduce the aggregation of CNTs [19–21]. The most commonly used approach is noncovalent coating with hydrophilic carboxylic acid groups by oxidizing acid treatment [22–24]. However, to the best of our knowledge, studies characterizing CNTs according to the dispersibility produced by different oxidizing acids have been scarcely performed.

The macrophages of the reticuloendothelial system (RES) cannot recognize nonopsonized nanoparticles circulating in the blood system, including CNTs [25]. Premature removal by the RES will inevitably hamper the absorption efficiency, resulting in a decrease of circulating half-life and reduced bioavailability at the intended delivery site [26–27]. Several strategies have been developed to modify particle surfaces to address this limitation. Among them, conjugation with poly(ethylene glycol) (PEG) is one of the most effective methods because PEG is highly hydrophilic and flexible [28–29]. Functionalization using a hydrophilic polymer with positively charged groups is another effective way to reduce opsonization by the RES and enhance the dispersion of particles by electrostatic repulsion [25,30]. Cellular entry and uptake of these carriers can be considerably enhanced by cationic modification and passive drug delivery to a tumor site due to high membrane binding avidity can be achieved.

In this study, SWCNTs conjugated with PEG and polyethylenimine (PEI), which contains amino groups, were synthesized (CNTs-PEG-PEI). The length of the SWCNTs was first shortened by ultrasonic scission in different strong acid solutions for improving the dispersion in water. Afterwards, PEG and PEI were grafted onto the CNTs. This functionalization was supposed to attenuate the premature removal and loss of nanocarriers, and also to improve the targeting to the tumor site. The physical and chemical properties of CNTs-PEG-PEI were systematically characterized and doxorubicin (DOX), one of the most potent anticancer drugs applied in chemotherapy, was used as a model drug to be loaded [31]. Drug loading capacity, in vitro release characteristics, the killing efficacy of modified CNTs on the MCF-7 cell line, and cellular uptake efficacy were further investigated to assess the advantage of the difunctionalized CNT carriers over unmodified CNTs. Furthermore, apoptosis induced by DOX-loaded CNTs was also analyzed. The results show that the SWCNTs were shortened by treatment with different acids, and the resulting carriers are well dispersed. The functionalized CNTs conjugated with PEG and PEI can be internalized into cells more efficiently, and release the loaded DOX in a pH value-dependent manner. They show effective growth inhibition towards tumor cells through inducing apoptosis.

## Experimental

### Chemicals and reagents

SWCNTs with high purity were purchased from Chengdu Organic Chemicals Co. Ltd., Chinese Academy of Sciences. Polyethyleneimine (PEI, *M*_w_ = 600 Da) was purchased from Aladdin Chemistry Co., Ltd. Doxorubicin hydrochloride (DOX) was purchased from Dalian Meilun Biological Technology Co., Ltd (Dalian, China). Cell culture medium RPMI 1640, fetal bovine serum (FBS), penicillin/streptomycin solution and fluorescent Hoechst 33342 were obtained from Beijing Solarbio Science & Technology Co., Ltd (Beijing, China). Trypsin–EDTA solution (0.25% trypsin with 0.02% EDTA) was supplied by Beyotime Biotechnology (Beijing, China). 3-(4,5-dimethylthiazol-2-yl)-2,5-diphenyl tetrazolium bromide (MTT) was purchased from Sigma-Aldrich (St. Louis, MO). NHS-PEG-COOH (*M*_w_ = 5 kDa) was purchased from Shanghai Ponsure Biotechnology Co., Ltd. 1-(3-Dimethylaminopropyl)-3-ethylcarbodiimide hydrochloride (EDC·HCl) and *N*-hydroxysuccinimide (NHS) were purchased from Beijing Solarbio Science & Technology Co., Ltd. *N*,*N′*-Carbonyldiimidazole (CDI) was purchased from Shanghai Macklin Biochemical Technology Co., Ltd. Dicyclohexylcarbodiimide (DCC) was purchased from Aladdin. Ultrapure water was produced with a Milli-Q water system. Dimethyl sulfoxide (DMSO) was produced from Tianjin Kemiou Chemical Reagent Co., Ltd. All other reagents were of analytical grade.

### Cell culture

The human breast cancer cell line MCF-7 used in this study was obtained from American Type Culture Collection (ATCC, Manassas, VA), and cultured in RPMI 1640 medium containing 10% (v/v) fetal bovine serum (FBS) and 1% penicillin–streptomycin at 37 °C in a humidified atmosphere with 5% CO_2_.

### Synthesis and functional modification of SWCNTs

SWCNT nanocarriers were synthesized and purified in a similar manner as described in [32]. Briefly, 300 mg of raw SWCNTs were suspended in 120 mL of strong acid solutions and ultrasonically treated for 1 h. Three different acid solutions were used to shorten SWCNTs, that is, H_2_SO_4_/H_2_O_2_ (1:1, v/v), HNO_3_, and H_2_SO_4_/HNO_3_ (1:3, v/v). Subsequently, the suspension was intensively stirred at 80 °C for 8 h. After that, the mixture was refluxed and diluted with distilled water, and then filtrated through a 0.22 µm PTFE microporous membrane. The product was collected and washed with deionized water to adjust the pH value to 6.8–7.2. These steps were repeated to remove the unacidified SWCNTs. The shortened CNTs were finally obtained by vacuum drying at 50 °C for 24 h. The resulting purified sample was denominated CNTs-COOH (CNTs).

The purified CNTs were afterwards conjugated with PEG and PEI. The typical procedure is as follows: An amount of 100 mg of CNTs-COOH was redispersed in 50 mL of distilled water and ultrasonically treated for 1 h. EDC·HCl (500 mg) and NHS (500 mg), used as catalyst, were added into the mixture under stirring at 45 °C overnight. The resulting suspension was then reacted with NHS-PEG-COOH (400 mg) at room temperature under stirring until the precipitate dissolved completely [33]. After dialysis for 2–3 days, the product CNTs-PEG was obtained by vacuum drying at 50 °C.

Subsequently, 50 mg of CNTs-PEG was redispersed in 150 mL of methanol and ultrasonically treated for 1 h. EDC·HCl and NHS were added under stirring at room temperature for 4 h. 200 mg of PEI was added and reacted with the CNTs-PEG solution under stirring for 24 h. Then, the precipitate was washed with methanol and deionized water alternately. The product was obtained by dialysis and vacuum drying successively as above. This difunctionalized CNT carrier was named CNTs-PEG-PEI.

### Characterization of nanocarriers

Different aqueous dispersions of SWCNTs were dispersed onto holey carbon film on copper grids. The sample morphologies were observed by transmission electron microscopy (TEM, JEM-200CX).

To determine particle size and zeta potential, a sample of 200 μL (with a concentration of 50 μg·mL^−1^) was dispersed in deionized water to a final volume of 3 mL. Particle size and zeta potential were measured using laser particle analysis (Malvern Zetasizer 3000HS). A volume-weighted Gaussian size distribution was fit to the autocorrelation functions to obtain the particle size and zeta potential values.

The atomic fractions of C, O, and N in the different SWCNTs samples were determined by X-ray photoelectron spectroscopy (XPS, Thermo Fisher Scientific). Fourier-transform infrared (FTIR) spectra in the range from 500 to 4000 cm^−1^ were recorded with a FTIR spectrometer (Nicolet IS10). X-ray diffraction (XRD) analysis was conducted using a BRUKER D8 X-ray diffractometer in the 2θ range of 0–100° at a scanning rate of 5°·min^−1^.

For atomic force microscopy (AFM) measurements, different SWCNT dispersions (ethanol/ultrapure water = 1:1) with a concentration of 0.01% were dripped on freshly cleaved mica and observed using an AFM (Dimension Icon, Bruker AXS).

### Drug loading and encapsulation efficiency

An amount of 50 mg of different SWCNT samples were suspended in DOX solution (1 mg·mL^−1^, 10 mL, dissolved in PBS, pH 7.4) and stirred at 37 °C for 24 h. After centrifuging, the precipitate was collected and washed with PBS. Finally, DOX-loaded samples were obtained by centrifugation and vacuum drying. Samples were prepared the day before the experiment and stored overnight at 4 °C.

The different DOX-loaded formulations were centrifugated 3–5 times for separating unencapsulated drugs. The supernatant liquid was stored for measuring the absorbance at 480 nm by UV–vis spectroscopy according to the preestablished standard curve. The drug loading efficiency (LE) and encapsulation efficiency (EE) were determined as: LE = weight of encapsulated DOX/weight of DOX-loaded carriers and EE (%) = weight of encapsulated DOX/weight of the total DOX·100%.

### In vitro drug release

The release of DOX from the nanocarriers was examined in PBS at pH 7.4 and pH 5.0 [34]. Different DOX-loaded samples (5 mL, 2 mg·mL^−1^) were transferred into a dialysis bag (MWCO 14000 Da) and sealed, and then suspended into PBS under constant shaking at a speed of 100 rpm at 37 °C in a glass beaker. At specific time intervals, 1.5 mL of supernatant was taken from the beaker. At the same time, the same volume of fresh PBS was supplied in the release medium. The concentration of released DOX in all samples was determined by measuring the UV absorbance. The cumulative release rate of DOX was calculated and the measurements were repeated for three times, averaging the results.

### Cell viability

The in vitro cytotoxicity of different blank nanomaterials and DOX-loaded samples toward MCF-7 cells was assessed by using the MTT assay. In brief, cells were seeded into 96-well plates at a density of 5 × 10^4^ cells per well and attached for 48 h. Then the culture medium was removed, and cells were incubated with serum-free medium containing DOX solutions (1–100 μg·mL^−1^, dissolved in PBS) or different CNT formulations with different DOX concentrations (1–100 μg·mL^−1^, dissolved in PBS) for 48 h. The cytotoxicity of the raw SWCNTs and of the corresponding blank carriers contained in the DOX-loaded formulations were also examined (1–200 μg·mL^−1^). After incubation, 20 μL/well of MTT solution (5 mg·mL^−1^ in PBS) was added, and the cells were further incubated for 4 h. Then the culture medium was removed, 100 μL/well of DMSO was added to replace, and the 96-well plates were agitated for 30 min, to completely dissolve the formazan crystals. The absorbance of the final solution was measured at a wavelength of 570 nm using a microplate spectrophotometer (SpectraMax iD5) to calculate the cell viability. Groups without treatment were used as control.

### Cellular uptake of DOX-loaded nanocarriers

The drug delivery in the intracellular matrix of free solution, CNTs-COOH, CNTs-PEG, and CNTs-PEG-PEI was quantitatively investigated by flow cytometry (FCM) to compare the uptake efficiency and intracellular accumulation of the different DOX-loaded nanocarriers. MCF-7 cells were cultivated into 12-well plates at 1 × 10^5^ cells per well under standard conditions overnight. After rinsing with PBS, free DOX (DOX solution) or DOX-loaded nanocarrier formulations in fresh serum-free RPMI 1640 medium with 20 μg·mL^−1^ of DOX were added into each well at 37 °C. After incubation for 12 or 24 h, the medium was removed from the wells, and the cells were trypsinized with trypsin–EDTA and washed with cold PBS for three times to remove the nanocarriers outside the cells. The cells were then collected and resuspended in 200 μL of PBS by centrifugation. The mean fluorescence intensity (MFI) was determined using FCM (BD Biosciences, SanJose), with 488 nm wavelength excitation and 590 nm wavelength emission, and untreated cells were used as blank.

Fluorescence observation of the cellular internalization was also conducted. In brief, cells were seeded into 24-well plates, after complete attachment, fresh serum-free medium containing free DOX or DOX-loaded nanocarrier formulations was added to replace the medium. After incubation for 12 h, the cells were rinsed with cold PBS for three times and fixed. The nuclei were subsequently stained by Hoechst 33342 for 30 min. A fluorescence microscope (Olympus, Tokyo, Japan) was used to detect and observe the fluorescence signal in cells.

Further, confocal laser scanning microscopy (CLSM) was utilized to observe the internalization. Cells were seeded into 6-well plates and incubated for 24 h. After co-incubation in 2 mL of medium containing free DOX or DOX-loaded nanocarrier formulations, the nuclei were afterwards stained with DAPI for 10 min, and the cells were fixed. For analysis, cells were imaged immediately using a confocal microscope (Leica TCS SP5, Leica Microsystems GmbH, Germany).

### Apoptosis assay

The apoptosis assay was performed using the Annexin V-FITC/propidium iodide (PI) Apoptosis Detection Kit. Cells were first seeded into 6-well plates and incubated for 24 h. Then the cells were treated with fresh serum-free medium containing free DOX or DOX-loaded nanocarrier formulations with a DOX concentration of 50 or 100 μg·mL^−1^ in the dark. The cell apoptosis after adding raw SWCNTs and corresponding blank carriers was also examined. After incubation for 48 h, the medium was removed, and cells were trypsinized and subsequently fixed overnight with ice-cold ethanol. Cells were then washed with PBS and centrifuged; the harvested cells were resuspended with 1 mL of loading buffer and transferred into a 15 mL tube. Following staining with 5 µL of Annexin V-FITC and PI, the cells were incubated for 15 min at 4 °C. The cell suspensions were then immediately analyzed by FCM, with 488 nm excitation wavelength and 575 nm emission wavelength. Groups without staining with Annexin V-FITC and PI were used as control. Cell morphology was also observed using the fluorescence microscope.

### Data processing and analysis

Data were given as mean ± standard deviation (SD), and mean values were considered significantly different when *p* < 0.05 (*) or *p* < 0.001 (***) using one-way analysis of variance (ANOVA).

## Results and Discussion

### Synthesis, functionalization and characterization of SWCNT-based nanocarriers

**Figure 1:**
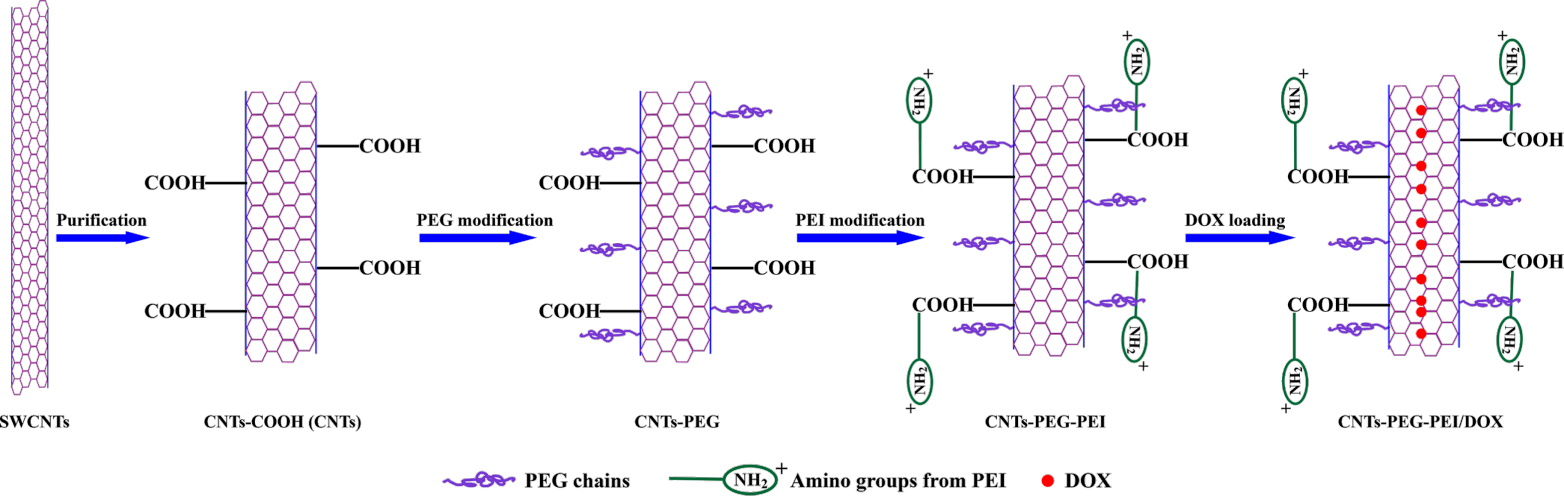
Schematic representation of CNTs-PEG-PEI nanocarriers and the drug-loading process.

Design and preparation process of the CNTs are described in Figure 1. Carboxylic acid-functionalized SWCNTs are oxidatively shortened, and the short CNTs are conjugated with PEG and PEI. The main intrinsic drawbacks of SWCNTs are the strong hydrophobicity and easy aggregation in aqueous media [9]. The raw SWCNTs are purified with concentrated acid to obtain carboxylated carbon nanotubes (CNTs-COOH), which significantly improves their water solubility [35–36]. NHS-PEG-COOH is a hydrophilic polymer that can graft firmly onto CNTs by non-covalent bonding. In contrast to covalent bonding methods, this non-covalent strategy is more facile and efficient, and will not affect the inherent optical properties of CNTs. The long hydrophilic chains of PEG and the amino groups from PEI make the CNTs much more soluble in aqueous solution and thus improve their biocompatibility [37]. Furthermore, surface modification with PEGylated agents or positively charged groups can protect the nanocarriers by providing a steric barrier from being recognized and captured by the mononuclear phagocyte system (MPS), and promote their cellular uptake and in vivo stability.

**Figure 2:**
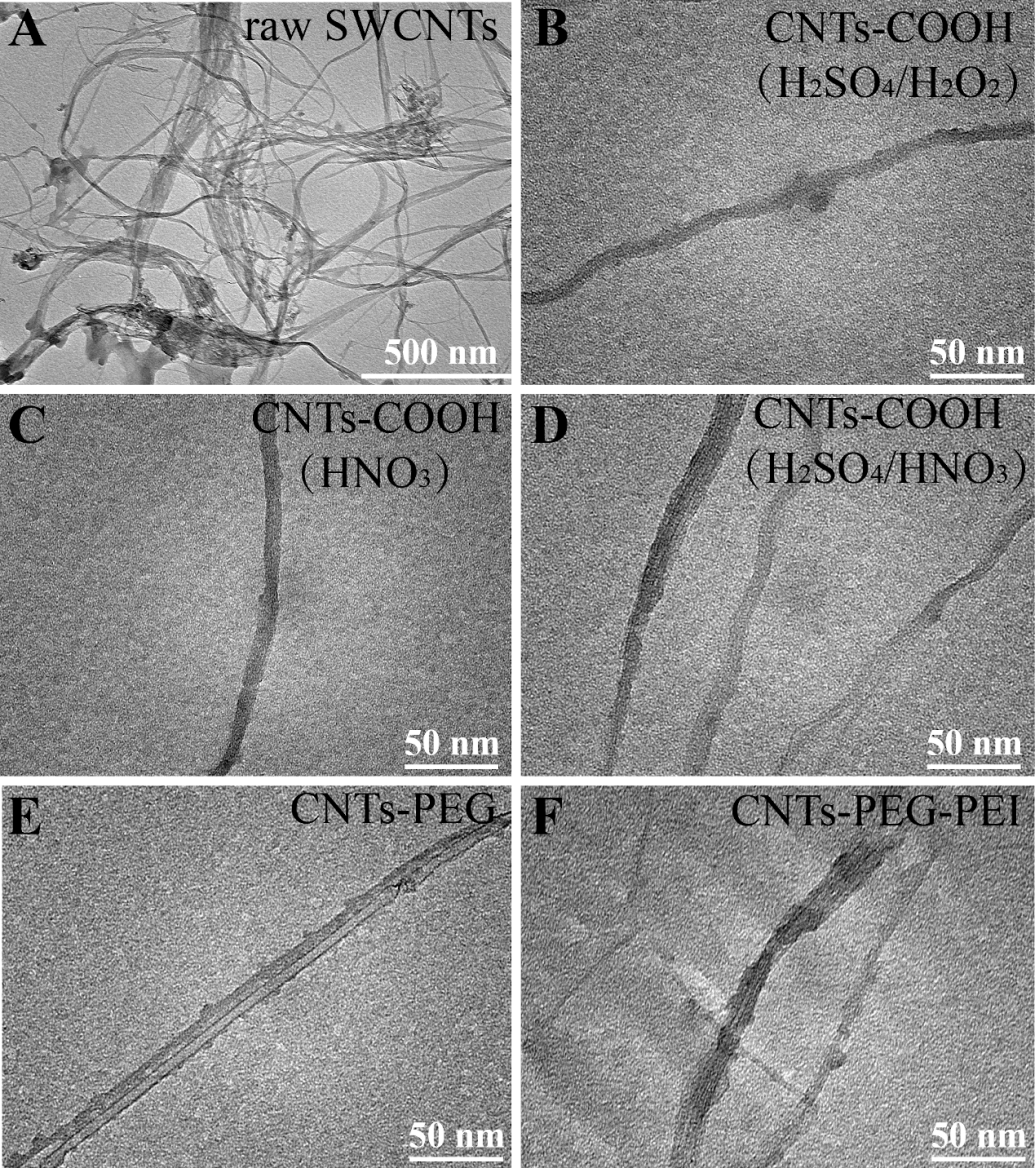
TEM images of (A) raw SWCNTs, (B–D) CNTs-COOH synthesized using different acid solutions: (B) H_2_SO_4_/H_2_O_2_, (C) HNO_3_, (D) H_2_SO_4_/HNO_3_, (E) CNTs-PEG and (F) CNTs-PEG-PEI.

**Figure 3:**
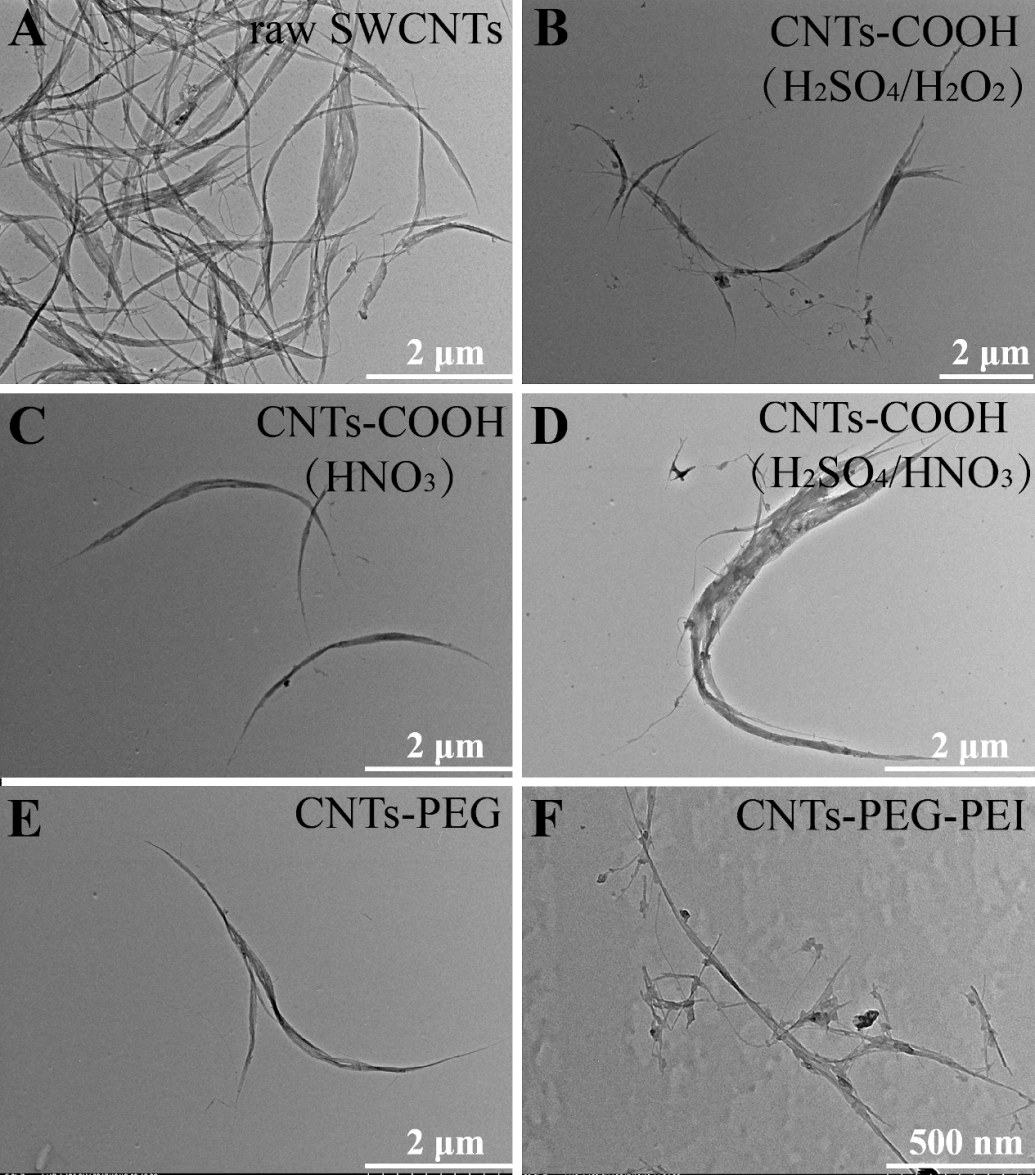
TEM images of (A) raw SWCNTs, (B–D) CNTs-COOH synthesized using different acid solutions: (B) H_2_SO_4_/H_2_O_2_, (C) HNO_3_, (D) H_2_SO_4_/HNO_3_, (E) CNTs-PEG and (F) CNTs-PEG-PEI.

TEM images of shortened and of functionalized CNTs are shown in Figure 2 and Figure 3 (with different magnifications), respectively. TEM images show that the raw SWCNTs are long curved aggregates, which appear to be a bundle of inhomogeneous aggregates consisting of many tubes (Figure 2A, Figure 3A). The length and dispersion state of raw and of acid-treated CNTs are quite different. After acid treatment, short CNTs exhibit a smaller tube length and a tubular structure with a hollow lumen, which is beneficial for the endocytosis of nanotubes. Three kinds of CNTs-COOH samples, produced using different acid solutions, are all dispersed into single tubes with smooth surface without aggregation, and no obvious difference in the morphological characteristics is observed (Figure 2B–D, Figure 3B–D). The tubular structure of CNTs-PEG and CNTs-PEG-PEI is rough, and some particles appear to be attached and distributed along the CNTs sidewalls, maybe indicating that PEG and PEI groups are conjugated onto nanotubes (Figure 2E,F, Figure 3E,F).

The results listed in Table 1 show that the average particle size (length) of the CNTs-COOH sample is distinctly smaller than that of the raw SWCNTs, and the sample treated with H_2_SO_4_/HNO_3_ mixed acid solution has the most even size distribution. The particle size of CNTs modified by PEG and PEI increases slightly. It should be noted that dynamic light scattering (DLS) is mainly suitable to determine spherical nanoscale samples, and the determination result or value of “size” is used to characterize their dimension. For nanoscale materials with tubular structure (or rod-like structure), such as SWCNTs, the determined size depends on the length as well as on the dispersion state. Although the numerical result obtained by size analysis does not represent the accurate length of the SWCNTs it is a good reference value for comparison.

**Table 1:** Particle size and zeta potential analysis results of different nanocarriers (*n* = 3). Data represent average ± standard deviation (SD).

Sample	Particle size (nm)	Polydispersity index (PDI)	Zeta potential (mV)

SWCNTs	2375.3 ± 276	1.0	−2.04 ± 0.9
H_2_SO_4_/H_2_O_2_	334.5 ± 72	0.638	−27.6 ± 1.9
HNO_3_	255.2 ± 54	0.489	−41.7 ± 2.3
H_2_SO_4_/HNO_3_	295.7 ± 77	0.228	−28.9 ± 4.6
CNTs-PEG	342.3 ± 85	0.348	+2.1 ± 3.7
CNTs-PEG-PEI	531.6 ± 133	0.442	+12.2 ± 1.8

The zeta potential of raw SWCNTs is nearly neutral, while those of all CNTs-COOH samples are significantly decreased and all below −25 mV, indicating that carboxy groups are present on the surface of CNTs and more charges are exposed at the outside of the nanotubes. After grafting of PEG and PEI, the zeta potential of CNTs sharply increases to above 0 mV, owing to the presence of amino groups, which also suggests the successful grafting of PEG and PEI. Considering the results, SWCNTs were purified and shortened with H_2_SO_4_/HNO_3_ in the subsequent experiments.

**Figure 4:**
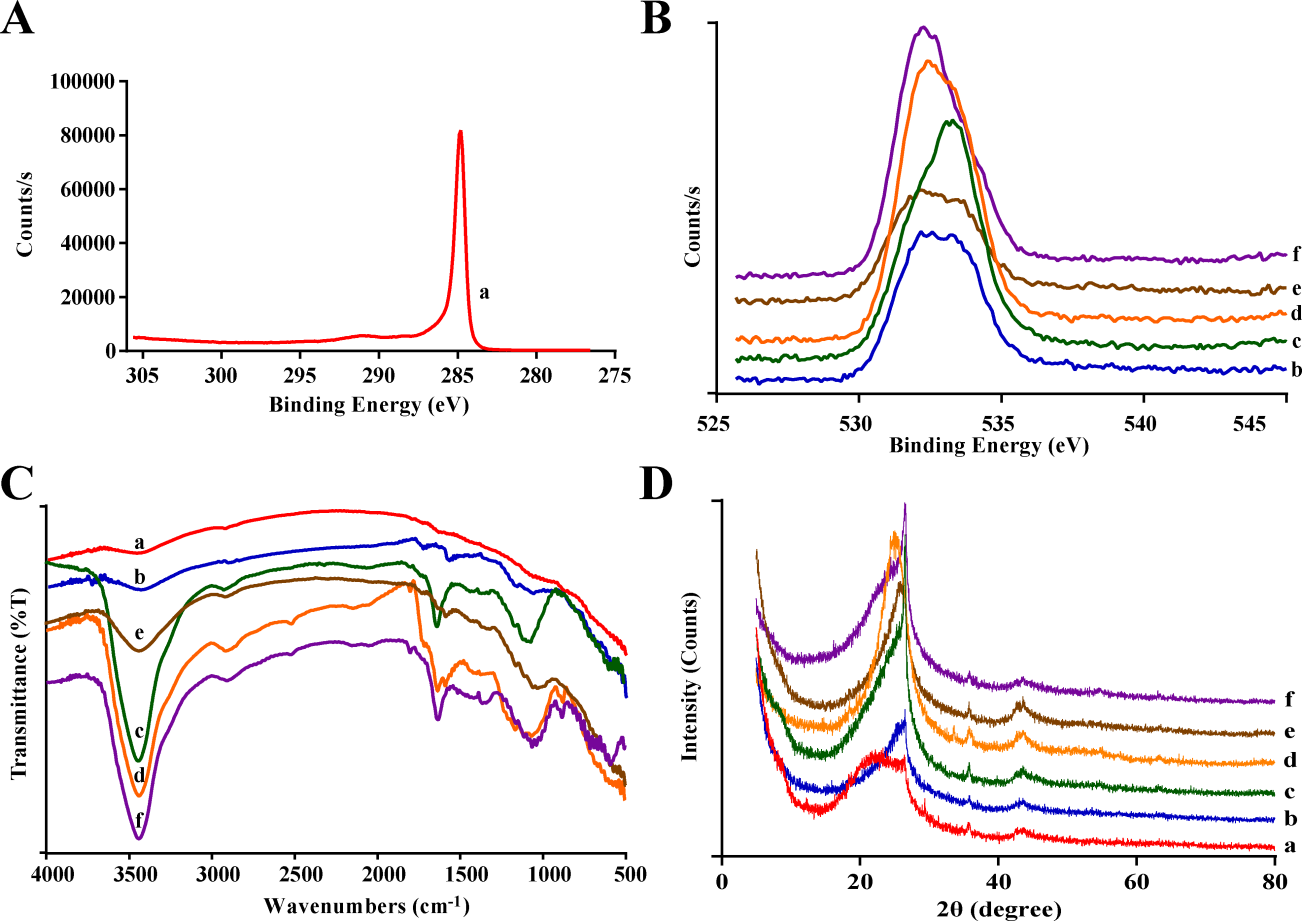
Characterization of different nanocarriers. (A, B) XPS spectra, (C) FTIR spectra, (D) XRD diffraction patterns (a: raw SWCNTs; b–d: CNTs-COOH synthesized using different acid solutions, b: H_2_SO_4_/H_2_O_2_, c: HNO_3_, d: H_2_SO_4_/HNO_3_; e: CNTs-PEG, f: CNTs-PEG-PEI).

The XPS spectra of different nanocarriers are shown in Figure 4A and Figure 4B. A C 1s peak with a binding energy of 285 eV appears for raw SWCNTs. In the CNTs-COOH samples a peak at 532.5 eV is detected, representing the simultaneous existence of double-bonded and single-bonded oxygen, which indicates the presence of carboxy groups on the surface of the CNTs. CNTs-PEG and CNTs-PEG-PEI exhibit a peak with a binding energy of about 531.8 eV, indicating the existence of OH groups.

As shown in Figure 4C, the weak peak of 3455 cm^−1^ in the FTIR spectrum of raw SWCNTs is characteristic for O–H. The absorption peaks at about 3434 cm^−1^ (indicating O–H) and 1630 cm^−1^ (denoting C=O) of the CNTs-COOH samples are both attributed to the carboxy groups, and the former becomes notably sharper. Moreover, the peaks at 1064 and 875 cm^−1^ indicate C–O and C–H, respectively, suggesting that the surface of the nanotubes contained more carboxy groups after the acidification reaction. Bands at 1361 and 750–600 cm^−1^ correspond to C–N and amide bond stretching vibrations, respectively, which demonstrates the successful conjugation of amino groups onto the surface of CNTs-PEG-PEI. XRD results (Figure 4D) show diffraction peaks of different carbon nanomaterials at 26° and 42°. In comparison with the raw SWCNTs, the diffraction peaks of CNTs-COOH samples become notably sharper. Furthermore, the diffraction patterns of the CNTs remain the same after conjugation with PEG and PEI, which indicate that the surface modification will not change the atomic structure of CNTs.

**Figure 5:**
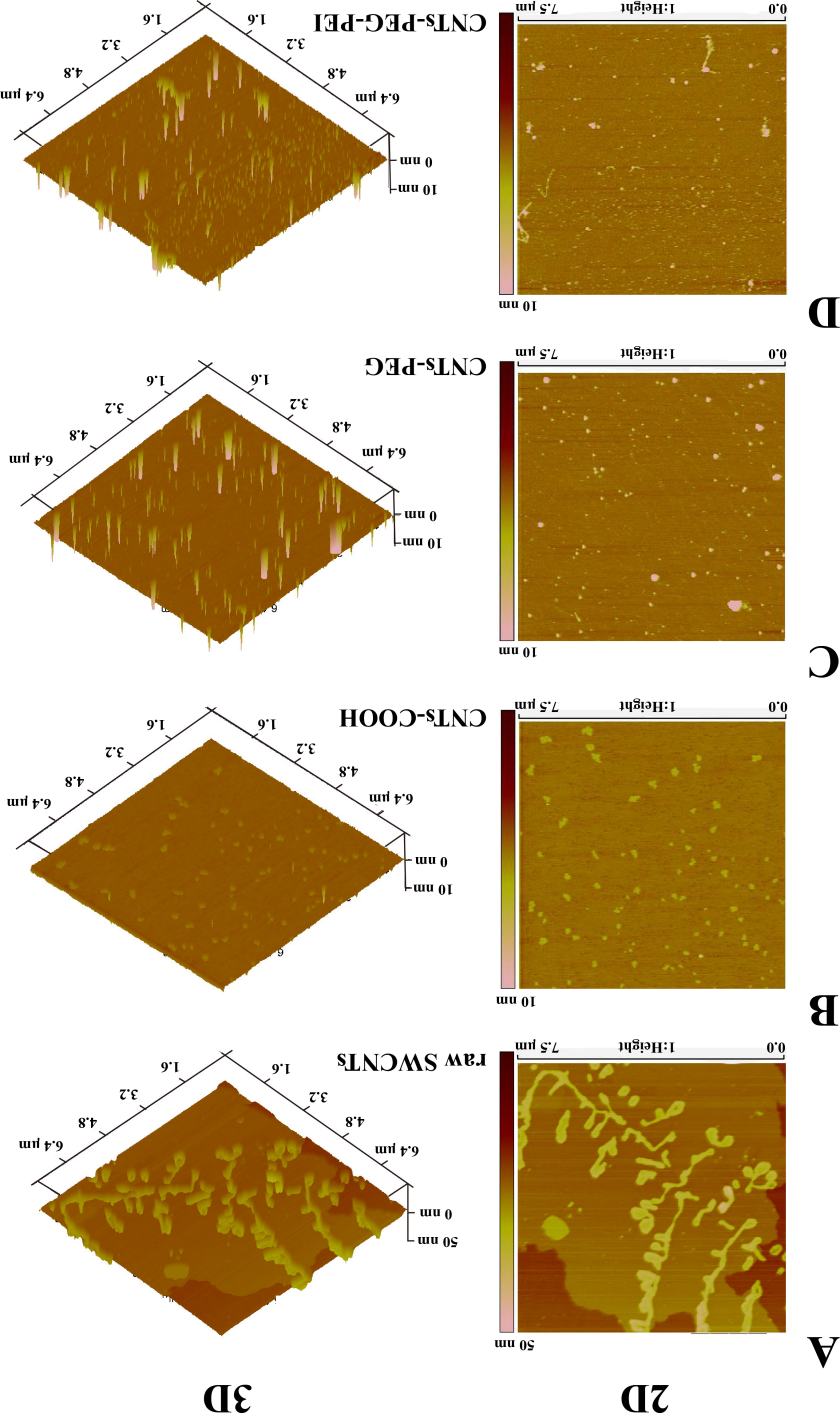
AFM images of (A) raw SWCNTs, (B) CNTs-COOH, (C) CNTs-PEG, and (D) CNTs-PEG-PEI.

The morphology of the different nanocarriers was observed using AFM after deposition on a mica substrate. The raw SWCNTs exhibit large bundles of aggregates, and many long tubes stay close together, which is consistent with TEM images. Compared with the raw SWCNTs (Figure 5A), CNTs-COOH, CNTs-PEG and CNTs-PEG-PEI samples are evenly dispersed on the substrate exhibiting tubular structures (Figure 5B–D), confirming their good dispersion. Moreover, their length is much shorter than that of the raw SWCNTs, also being consistent with the TEM observations. The above results prove that the raw SWCNTs were shortened by the acid treatment, together with an improvement of the dispersion state. Thus, highly stable dispersions of individual CNTs can be obtained by acidification and functionalization. All the above results demonstrate that the raw SWCNTs are shortened and functionalized while retaining their spectroscopical properties. The carboxylated and the functionalized CNTs are individually dispersed in aqueous solution, suitable for further biological studies [38].

It should be noted that in a former report, PEG-grafted hyperbranched polyethylenimine (PEG-*g*-PEI) was first synthesized by a carbodiimide reaction. It acted as a hydrophilic biocompatible block copolymer for the preparation of PEG-PEI-functionalized CNTs [30]. In this study, the carboxylated CNTs was conjugated with PEG groups after acidification, and subsequently modified by PEI. Although the functionalization approach and decoration sequence were different, the characterization results show that both approaches are feasible synthetic routes.

### Drug-loading efficiency

The obtained values of drug loading efficiency (LE) and encapsulation efficiency (EE) listed in Table 2 show that the shortened CNTs after acidification treatment can load DOX more efficiently than the raw SWCNTs. Both LE and EE are increased. Besides, it can be found that there is no significant change of LE and EE of DOX-loaded CNTs-PEG and CNTs-PEG-PEI, suggesting that the drug loading capacity of CNTs will not be affected by the surface conjugation with PEG and PEI. The enhancement of drug loading can be explained by the fact that the outer surface of CNTs-COOH is more hydrophilic than that of raw SWCNTs due to the presence of carboxy groups [39]. Therefore, hydrophilic DOX molecules could be loaded by CNT carriers through locating in the internal hollow space or through adsorbing on the sidewalls of the nanotubes. Also, the sidewalls of CNTs became more hydrophilic further after modification with PEG or PEI [40]. It should be noted that LE and EE of CNT carriers in this study are higher than those of other nanocarriers such as mesoporous silica nanoparticles (MSNs) or liposomes [39].

**Table 2:** LE and EE (%) results of different nanocarriers.

Sample	LE (μg/mg)	EE (%)

SWCNTs	9.5	5.6
H_2_SO_4_/H_2_O_2_	139.1	74.6
HNO_3_	77.3	69.1
H_2_SO_4_/HNO_3_	172.5	70.3
CNTs-PEG	168.0	76.0
CNTs-PEG-PEI	176.9	73.3

### In vitro drug release

**Figure 6:**
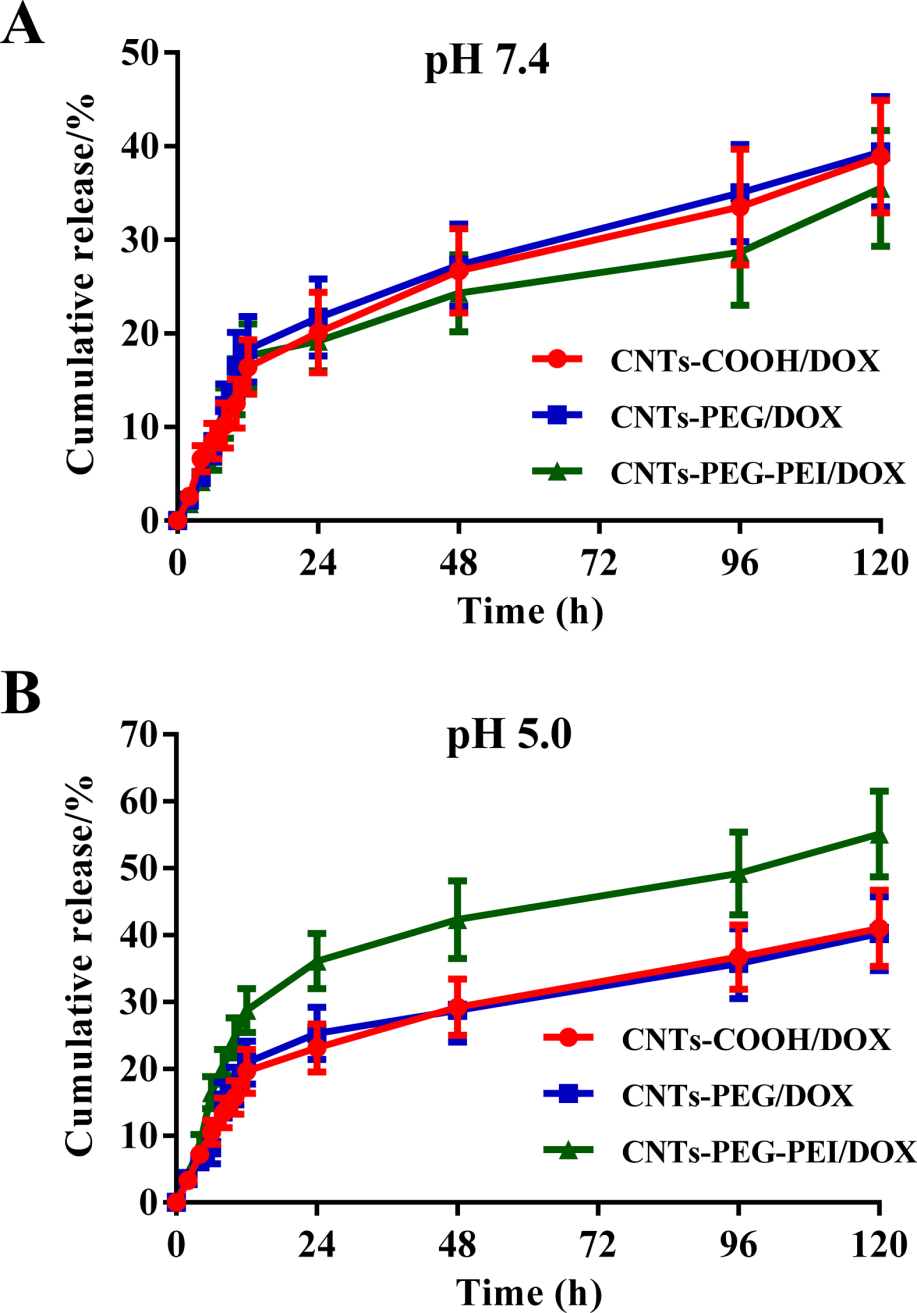
Cumulative release profiles of DOX from different nanocarriers in PBS at (A) pH 7.4 and (B) pH 5.0 (*n* = 3).

Due to the encapsulation in the nanocarriers after drug loading, a premature leakage of drug molecules is prevented, leading to a sustained release. Here, the release of DOX from CNT carriers was investigated in PBS at different pH values. Figure 6 shows the release curves of DOX from different drug-loaded CNTs. CNTs-COOH and CNTs-PEG exhibit a gradual release of DOX in PBS at pH 7.4, through which approximately 40% of DOX is released after 120 h. The cumulative release of DOX from CNTs-PEG-PEI is slightly lower [39] (Figure 6A). Under acidic conditions (pH 5.0), an accelerated release of DOX from CNTs-PEG-PEI is observed, with a cumulative release of more than 50% after 120 h (Figure 6B). This pH-dependent release of DOX loaded by CNTs-PEG-PEI can be attributed to a protonation effect resulting from the conjugation with positively charged amino groups. The encapsulation or detachment of drug molecules largely depends on their electrostatic interaction with the nanocarriers. With increasing acidity, the protonated amino groups can attenuate electrostatic attraction between DOX molecules and CNT carriers gradually. Thus, DOX molecules loaded within the tubes or absorbed onto the surface of CNTs at neutral pH values tend to be released more rapidly [41].

In view of the difference between the pH values of healthy blood and of tumor cells where the CNT carriers accumulate after endocytosis, especially in endosomes and lysosomes, this pH-dependent release will benefit the expected antitumor treatment. CNTs-PEG-PEI/DOX remains relatively stable when circulating in normal tissues. The DOX molecules are retained in the carriers and subsequently rapidly released from nanotubes at the targeted tumor sites with a slightly acidic environment [42].

### Cell viability

**Figure 7:**
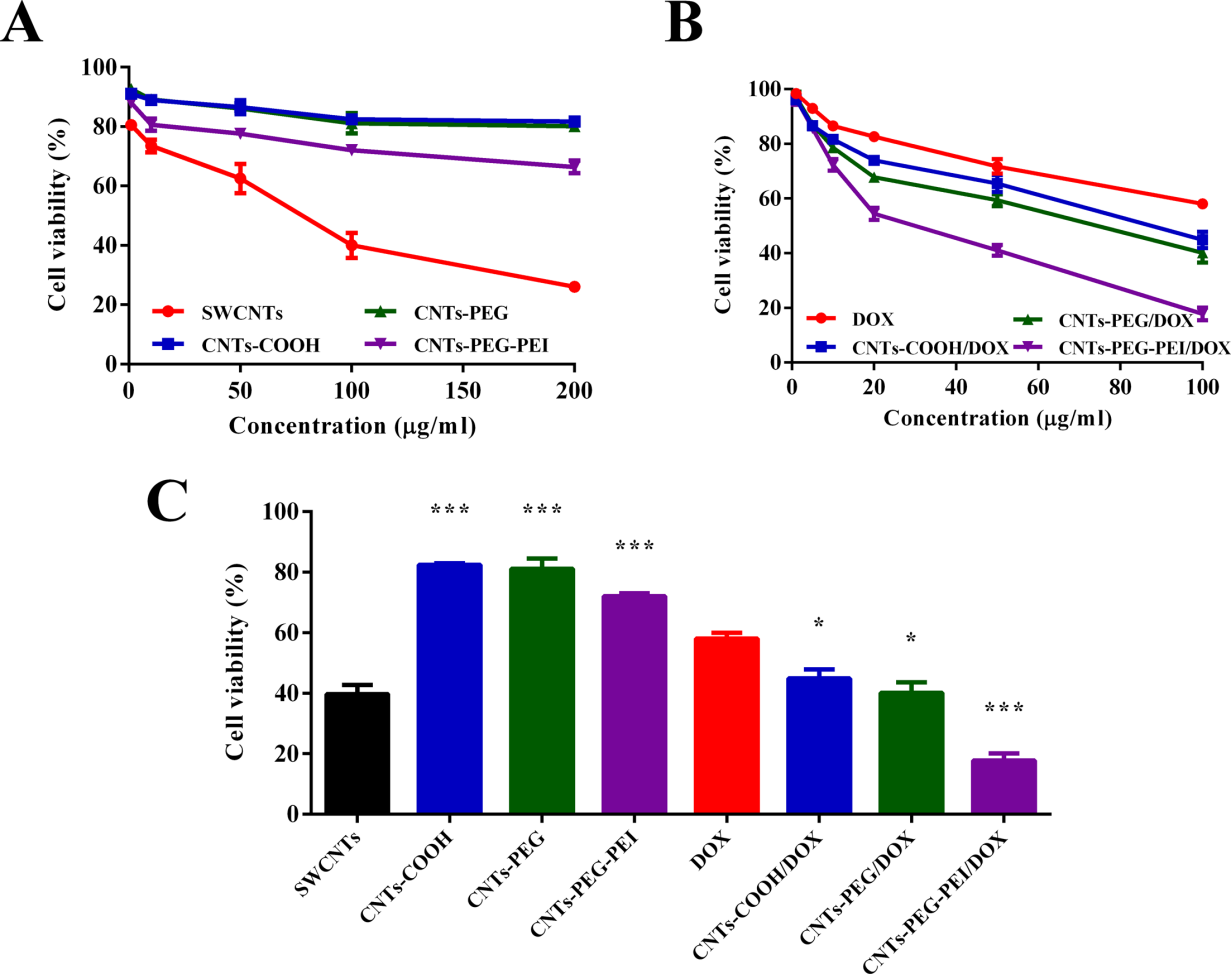
Cytotoxicity of (A) different blank carriers and (B) DOX-loaded formulations against MCF-7 cells after 48 h as a function of the concentration. (C) The cell viability results of different samples with a concentration of 100 μg·mL^−1^ (*n* = 3). ANOVA was used for statistical analysis.

Cytotoxicity assessment results of different blank carriers and DOX-loaded nanocarrier formulations are shown in Figure 7. The IC_50_ values of the DOX samples were calculated and listed in Table 3. The results show that the cytotoxic effect of raw SWCNTs is significantly higher than that of the other three materials. For instance, the viability of the cells treated with raw SWCNTs at a concentration of 200 μg·mL^−1^ is 26.2% after 48 h of incubation, while CNTs-COOH and CNTs-PEG show cell viability values greater than 80% (Figure 7A). The viability of cells treated with CNTs-PEG-PEI drops to about 66.7%, slightly lower than that of CNTs-COOH and CNTs-PEG, owing to the high number of positively charged amino groups on their surface, which leads to higher toxicity [6]. The IC_50_ values of the different carriers also suggest that the carboxylated and functionalized CNTs are more biocompatible. This result indicates that acidification treatment and the resulting enhancement of water dispersion yields more biocompatible SWCNT nanocarriers with low cytotoxicity.

**Table 3:** IC_50_ values of different SWCNT materials and DOX-loaded formulations against MCF-7 cells (*n* = 3). Data represent average ± standard deviation (SD).

Sample	IC50 (μg·mL^−1^)

raw materials	

SWCNTs	67.37 ± 12.26
CNTs-COOH	633.0 ± 78.2
CNTs-PEG	577.2 ± 60.3
CNTs-PEG-PEI	283.8 ± 36.3

DOX-loaded samples	

free DOX	164.0 ± 24.5
CNTs-COOH	91.15 ± 11.42
CNTs-PEG	65.29 ± 8.17
CNTs-PEG-PEI	27.14 ± 4.65

All DOX formulations exhibit effective anticancer activity against MCF-7 cells, and the cytotoxicity is observed to be dose-dependent (Figure 7B). It is noted that DOX-loaded CNT carriers show an enhanced inhibitory effects toward MCF-7 cells in comparison with free DOX. For example, the IC_50_ value of DOX-loaded CNTs-COOH, CNTs-PEG and CNTs-PEG-PEI groups are decreased by a factor of 1.80, 2.51, and 6.0, respectively, in comparison with that of free DOX. The comparison of cell viability of different samples (with concentrations up to 100 μg·mL^−1^) is shown in Figure 7C. The highest antitumor effect shown by difunctionalized CNT carriers may be the result of enhanced binding to the negatively charged tumor cells due to the electrostatic attraction, and the improved cellular uptake, which needs to be further clarified in a following study.

### Cellular uptake

**Figure 8:**
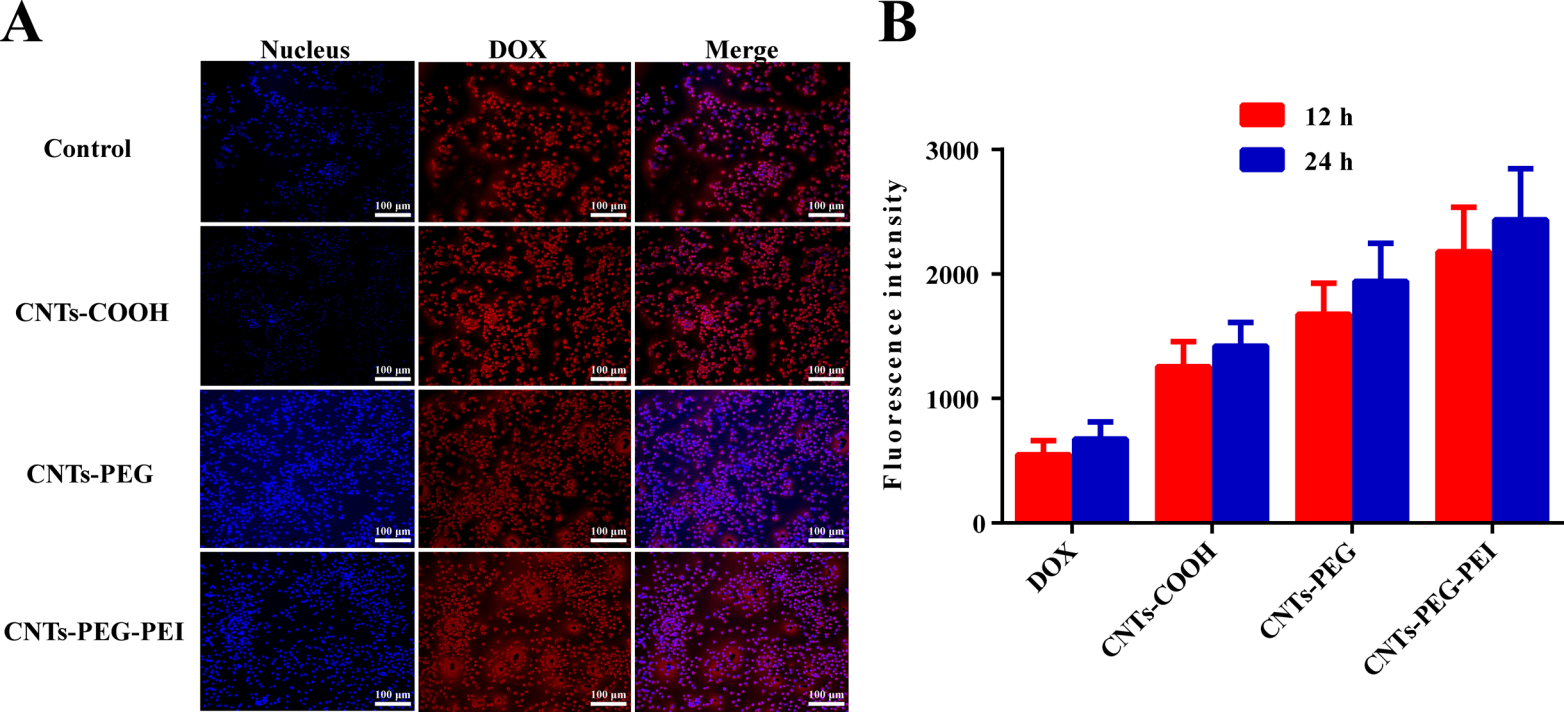
(A) Fluorescence images of MCF-7 cells after treatment with free DOX (control) and different DOX-loaded nanocarrier formulations for 12 h. (B) Fluorescence intensity of free DOX and different DOX-loaded nanocarrier formulations (*n* = 3). Student *t*-test was used for statistical analysis.

Efficient cellular uptake and intracellular drug release are decisive factors for drug delivery systems regarding therapeutic effects. Fluorescence images were recorded and compared to analyze the cell internalization and subsequent drug release of the different CNT formulations. The fluorescence intensity was further measured by FCM. The results are shown in Figure 8.

The results show that the fluorescence intensity detected in cells treated with different DOX-loaded CNTs was markedly increased both after 12 and 24 h in comparison with those incubated with free DOX (Figure 8A). The free drug molecules enter cells only by mobility or diffusion, while the individually dispersed nanotubes are more easily internalized by cells via direct penetration and endocytosis [38,43]. This can explain the above results. The fluorescence intensity of CNTs-PEG/DOX and CNTs-PEG-PEI/DOX increases further (Figure 8B). This is attributed to the fact that PEGylation and amino functionalization could be significant in facilitating the dispersion of nanotubes and decreasing their aggregation, which protects the CNT carriers from interaction with plasma protein components. This enhanced stability will favor attachment and interaction of carriers and cells [39]. Moreover, the promoted binding affinity with tumor cells provided by the positively charged surface of nanotubes will lead to enhanced cellular uptake and targeted delivery of CNT carriers [39].

**Figure 9:**
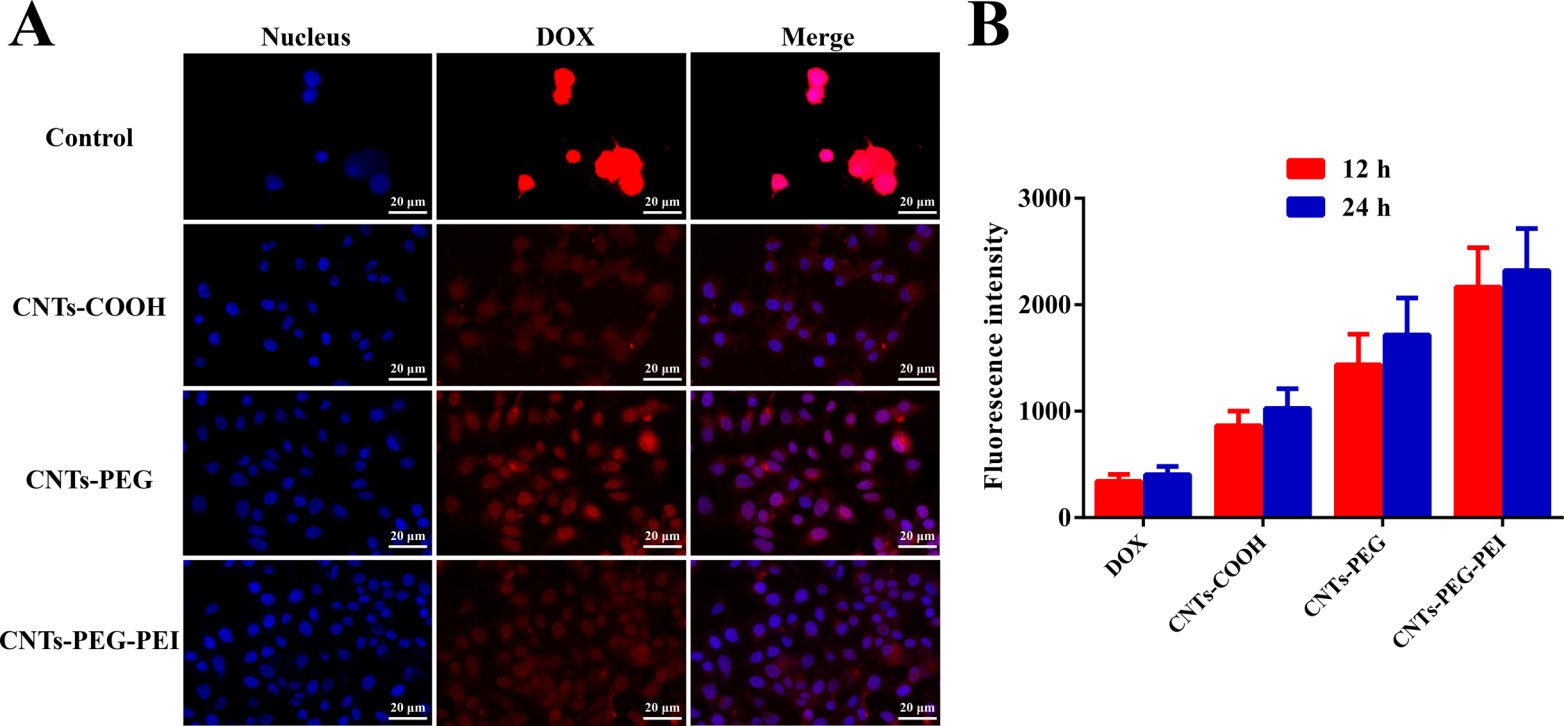
(A) Confocal microscopy images of MCF-7 cells after treatment with free DOX (control) and different DOX-loaded nanocarrier formulations for 12 h. (B) Quantitative fluorescence intensity of free DOX and different DOX-loaded nanocarrier formulations (*n* = 3). Student *t*-test was used for statistical analysis.

The intracellular co-localization results by CLSM are shown in Figure 9A. Large fluorescence signals representing DOX-loaded carriers are in the cytoplasm around the nucleus, suggesting that the cells have ingested the different nanocarriers and DOX was released into both cytoplasm and nucleus. The drug released in the cytoplasm can afterwards enhance the anticancer activity through injury of the nucleus or mitochondrial injury mechanisms. Furthermore, the fluorescence signal within cells treated with CNTs-PEG-PEI/DOX is higher than that of other groups (Figure 9B), which further illustrates the more efficient cellular uptake and delivery capacity of difunctionalized CNT carriers.

### Effects of DOX-loaded carriers on apoptosis in MCF-7 cells

**Figure 10:**
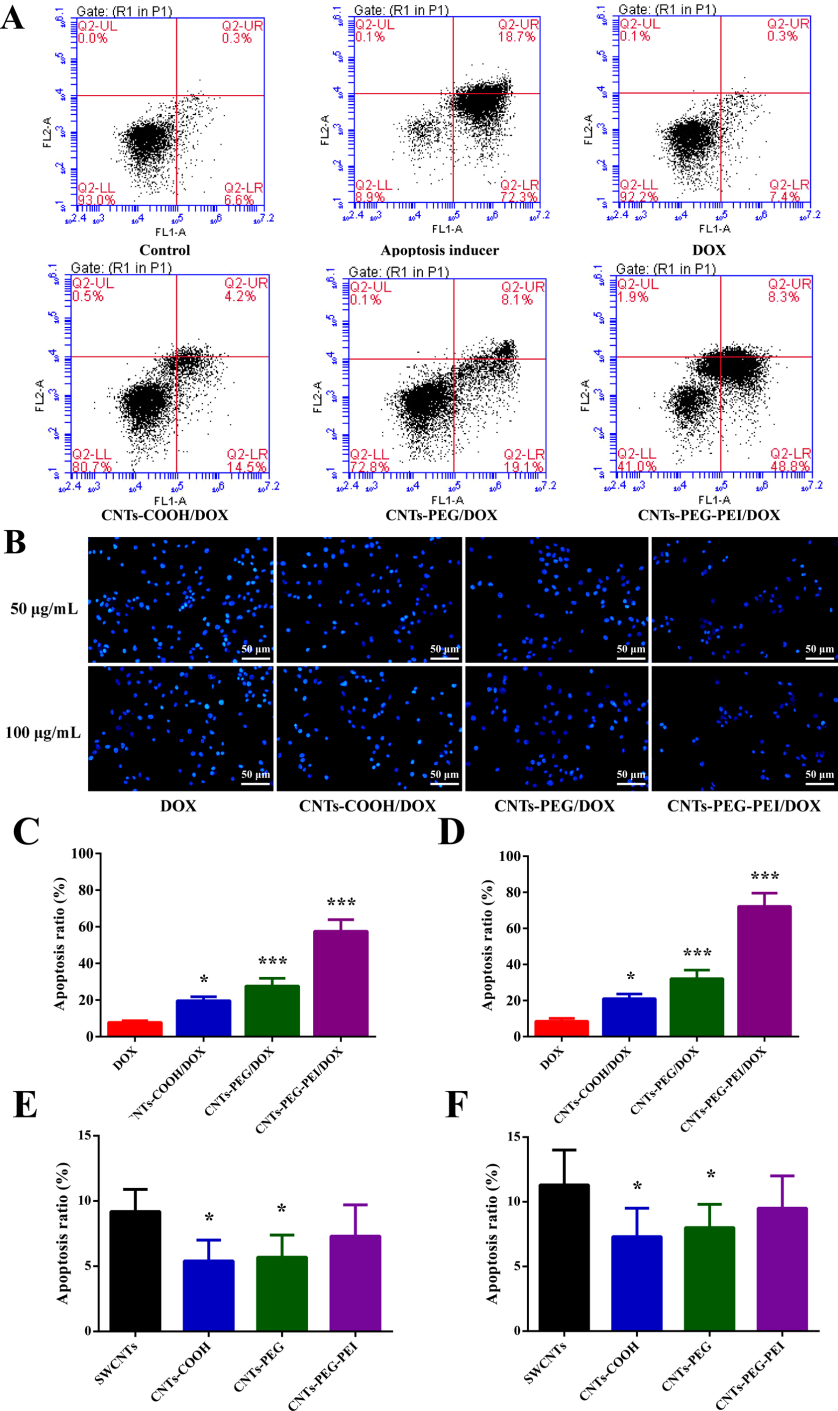
Effects of free DOX, different DOX-loaded nanocarrier formulations, and blank carriers on apoptosis in MCF-7 cells. (A) FCM scatter diagrams of MCF-7 cells treated for 48 h. (B) Fluorescence images of apoptotic cells. Quantitative apoptosis ratios: (C) DOX equivalent concentration 50 μg·mL^−1^; (D) DOX equivalent concentration 100 μg·mL^−1^; (E) blank carrier equivalent concentration 50 μg·mL^−1^; (F) blank carrier equivalent concentration 100 μg·mL^−1^ (*n* = 3). ANOVA was used for statistical analysis.

To clarify the antitumor mechanism of drug-loaded CNT carriers, the apoptosis of MCF-7 cells after treatment with the different nanocarrier formulations was investigated. Results from negative and positive control groups indicate that the state of MCF-7 cells is normal (Figure 10A). In most of the cells, apoptosis is induced after treatment with apoptosis inducer, and very few cells are necrotic (Figure 10B). Furthermore, the free DOX sample shows the highest number of scatter points in the lower left quadrant (Q3), whereas the corresponding distribution percentages in this area of the different nanocarrier formulations groups are decreased. Accordingly, cell numbers scattered in the upper right (Q2) and lower right (Q4) areas are gradually increased. It is well known that the quadrants Q2 and Q4 correspond to late apoptosis and early apoptosis, respectively.

It is not effective to induce cell apoptosis with free DOX. The apoptosis ratios after treatment with DOX-loaded CNTs are notably higher than that after treatment with free DOX (7.8%), with rates of 19.6%, 27.6% and 57.5% for CNTs-COOH, CNTs-PEG and CNTs-PEG-PEI, respectively (DOX equivalent concentration 50 μg·mL^−1^, 48 h after incubation). Also, apoptosis ratios measured at a DOX concentration of 100 μg·mL^−1^ are all slightly higher for each formulation (Figure 10C,D). Living cell fluorescence images also confirm these results (Figure 10B). Furthermore, the raw SWCNTs can induce cell apoptosis with ratios of 9.2% and 11.3% at 50 and 100 μg·mL^−1^, respectively. The apoptosis ratios of carboxylated or functionalized SWCNTs are lower (Figure 10E,F). It can be concluded that drug-loaded CNT carriers can cause the death of cancer cells through apoptosis after delivering chemotherapeutic drugs into the cells [44–45]. All the above experimental results reveal that CNTs-PEG-PEI carriers have advantages over CNTs-COOH and CNTs-PEG, that is, a rapid drug release at low pH values, a higher cell internalization efficiency, and an enhanced killing ability toward tumor cells via apoptosis.

In a former report, CNT carriers modified with PEG or PEG-PEI were evaluated thoroughly in term of DOX loading and release [30]. The investigation results confirmed that pH value and DOX concentration had an influence on the loading parameters. Under optimal conditions, CNTs-PEG-PEI carriers had a high EE (>90%). Furthermore, the LE of CNTs-PEG and CNTs-PEG-PEI was increased with an increase in the DOX concentration. The respective values in this study were lower than those of the above report, as experimental conditions were not carefully optimized. In addition, CNTs-PEG-PEI carriers in [30] exhibited sustained release at pH 7.4, and responsive release under relatively acidic condition, which was similar to our results. In this study, a series of experimental observations with MCF-7 cells were conducted and the results verify the delivery capacity and in vitro antitumor activity including induction of apoptosis, especially for CNTs-PEG-PEI carriers. These were not reported previously.

## Conclusion

In this study, CNTs conjugated with PEG and PEI for delivery of antitumor drugs were synthesized. The raw SWCNTs were shortened by acidification with different acid solutions. Considering the results regarding particle size and zeta potential, H_2_SO_4_/HNO_3_ was subsequently selected to produce carboxylated CNTs. Characterization results confirm the successful grafting of PEG and PEI onto CNTs, and different CNT nanocarriers can load DOX with high LE and EE. Compared with CNTs-COOH and CNTs-PEG, CNTs-PEG-PEI exhibited excellent performance as nanocarriers, including accelerated drug release under acidic conditions, enhanced cellular uptake efficiency and improved in vitro antitumor effect. It was further shown that DOX loaded by the different CNT carriers in the internal hollow space or adsorbed on the sidewalls can be effectively delivered into tumor cells and afterwards gradually released. In addition, due to the good dispersion state of single nanotubes and the comparably higher affinity with tumor cells, the increased uptake of CNTs-PEG-PEI/DOX by MCF-7 cells will lead to more efficient cell internalization and accumulation. The subsequent drug release and diffusion within the cytoplasm can induce apoptosis. The results suggest that CNTs-PEG-PEI may be developed to be a novel nanoscale delivery system of chemotherapeutic drugs for cancer therapy.
